# Comparison of cultures and 16S rRNA sequencing for identification of bacteria in two-stage revision arthroplasties: preliminary report

**DOI:** 10.1186/s12891-016-0991-1

**Published:** 2016-03-25

**Authors:** Przemysław Bereza, Alicja Ekiel, Aleksandra Auguściak-Duma, Małgorzata Aptekorz, Iwona Wilk, Damian Kusz, Piotr Wojciechowski, Aleksander L. Sieroń, Gayane Martirosian

**Affiliations:** Department of Orthopaedic and Traumatology, Medical University of Silesia, School of Medicine in Katowice, Ziołowa 45/47, Katowice, 40-635 Poland; Department of Medical Microbiology, Medical University of Silesia, School of Medicine in Katowice, Katowice, Poland; Department of General, Molecular Biology and Genetics, Medical University of Silesia, School of Medicine in Katowice, Katowice, Poland; Department of Histology and Embryology, Medical University of Warsaw, Warsaw, Poland

**Keywords:** Prosthesis-related infections, Reoperation, Biofilms, Sonication, 16S Ribosomal RNA

## Abstract

**Background:**

The use of a prefabricated spacer in two-stage revision arthroplasty remains one of the few surgery strategies for infected-joint arthroplasty treatment, despite the many unidentified microorganisms in the infected joint replacements reported in some recent studies. The aim of this prospective survey was to investigate if the sonication followed by polymerase chain reaction (PCR) can improve bacterial identification on the surfaces of prefabricated spacers and if the systemic laboratory mediators of infection and positive microbiological results can take a role of predictive factors of infection and clinical failures in 2-years follow-up.

**Methods:**

Thirteen patients with prosthetic joint infection were investigated. Bacterial culture and deoxyribonucleic acid (DNA) sequencing were used to detect bacteria on the surface of prefabricated spacers removed during the second stage of revision arthroplasty. The results of pre- and intraoperative culture and DNA sequencing were compared. Minimum follow-up was 2 years.

**Results:**

The result of tissue cultures in second-stage revision arthroplasties revealed positive results in 15 % of patients with Coagulase-negative Staphylococci (CNS) growth. Bacterial DNA was found in over 90 % of patients with negative synovial fluid culture. Positive PCR results revealed potential pathogenic bacteria and species of human and environmental microflora with low virulence. Clinical failures at final follow-up were recorded in 2 (16.6 %) patients.

**Conclusion:**

The lack of clinical signs of infection, negative culture of preoperative joint aspirate, and intraoperative specimens do not exclude the presence of bacteria on the surfaces of spacers. The positive results of sonication and molecular tests should be interpreted as real pathogenicity factors in the light of the clinical and laboratory data, especially for patients with immunodeficiency. We confirmed our previous results that sonication followed by PCR and sequencing improved bacterial identification.

## Background

One of the most severe complications after joint replacement is prosthetic joint infection (PJI). The risk of PJIs occurs in 3.2–7 % of patients after revision arthroplasties [1]. Currently, the two-stage exchange arthroplasty is the preferred method of treating chronic PJI [2–6]. The use of pre-formed spacers, which are implantable devices indicated to temporarily replace a prosthesis in a septic revision procedure, allow local antibiotic administration and maintains patient mobility between stages. The usage of prefabricated spacers limited the risk of spacer fracture and facilitate procedure of second-stage arthroplasty [6]. A recent study revealed 73 % successful two-stage revision arthroplasties [7]. One-stage exchange arthroplasty is advocated by some surgeons with comparable outcomes to two-stages surgeries. Extensive and radical soft tissue debridement and removal of the biofilm covered prosthesis are the main goals of one-stage revision arthroplasty [8]. One-stage procedure has not been commonly used in PJI because of uncertainty of total bacterial eradication, which could be the cause of surgeon concern about recurrent infection. In most cases successful eradication of bacteria is considerably enhanced by correct identification of the pathogen using tissue and joint fluid culture. Recent studies revealed that 2–36 % of microorganisms in the infected joint replacements were not identified [7, 9–12].

In the present study we investigated bacterial species in supposedly healed PJI patients during second-stage exchange arthroplasties. This study was designed to detect and/or isolate bacteria presented on the surfaces of the prefabricated antibiotic-loaded spacers during the second stage revision surgery. In our previous study we identified the bacteria on removed loosened prosthesis with the use of sonication and PCR (polymerase chain reaction) procedures in patients without elevated inflammatory markers. Our clinical interest to perform this study was to find the answer to following questions: if the supposedly healed PJI should be considered as aseptic without the fear for reimplantation and if failures could be predictable in some cases?

The aims of the present study were: (1) to show that sonication followed by PCR can improve bacterial identification on the surfaces of spacers used as a temporary implant eluting antibiotic in the site of periprosthetic infection, (2) to prove that the normalization of laboratory markers does not exclude silent persistent infection and the presence of bacteria on spacer surfaces, and (3) to determine if laboratory markers of infection and culture results were related to failure at 2-years follow-up.

## Methods

### Patients

Thirteen patients (7 women and 6 men) aged 50–84 years (mean age 69.2), before second-stage surgery of PJI, were qualified to this study. We recruited 13 patients (4 with hip and 9 with knee diagnosed joint infection) attending the Department of Orthopaedic and Traumatology, Medical University of Silesia, School of Medicine in Katowice, Poland. The average period between the first and second stage of revision arthroplasty was calculated at 153.1 days (approximately 5 months). Minimum follow-up on these 13 patients was 2 years (mean, 32 months; range, 25–36 months). No patients were lost to follow-up*.* Failure was considered as clinical failure in case of persistent or recurrent local signs of infection confirmed by positive culture results requiring long-term antimicrobial therapy and/or reoperation. Clinical examination, radiological assessment and determination of inflammatory markers were conducted between stages of revision arthroplasties.

This study was approved by the Ethics Committee of the Medical University of Silesia in Katowice, Poland (No. of Decisions: KNW/0022/KB1/160/III/11/12 and KNW/0022/KB1/160/V/11/12). The patients were informed about the aim and methods of this study and gave written informed consent. Also, consent for publication of raw data was obtained from study participants.

Inclusion criteria were: patient was operated in our Department awaiting second-stage revision arthroplasty of hip or knee, primary qualified as PJI, or highly suspected as PJI, based on the established criteria [2, 3].

Exclusion criteria were: antibiotics administration 2 weeks before revision arthroplasty, other established infection sites in the organism, rheumatoid arthritis, immunosuppression and/or chemotherapy, and lack of patient consent for participation in the study.

### Surgical management

Patients were qualified to the two-stage revision arthroplasties. The first stage included removal of the components of prosthesis and/or cement, debridement of necrotic and granulation tissue, and the implantation of a prefabricated antibiotic-loaded cement spacer with gentamicin (Spacer-K and Spacer G manufactured by Tecres S.P.A., Verona, Italy). The standard prophylactic antibiotic therapy was cefamandole. In case of allergy to beta-lactam antibiotics, vancomycin or clindamycin was administered. In case of positive intraoperative specimen after the first stage, antibiotic therapy was adjusted according to the microbiological findings. The empirical or targeted antibiotic therapy was administered for 6 weeks. After normalization of infection markers and lack of periprosthetic infection clinical symptoms, the next step of exchange arthroplasty was undertaken. In the second stage of revision arthroplasty, the temporary spacer was removed and revision hip or knee system or arthrodesis (patient 13) was applied. The arthrodesis of the knee joint was performed due to a general medical condition of the patient that precluded major surgery and high risk of reinfection.

### Microbiological and Molecular methods

Intraoperative tissue samples taken during the first stage of exchange arthroplasty (removal of the implants, debridement, and spacer implantation) were cultured. Before cutting the pseudocapsule during the second stage of the operation (removal of spacers and implantation of a new prosthesis or, eventually, arthrodesis), the joint was aspirated. During the surgery, at least 3–6 tissue specimens for microbiological culture were also taken. Specimens were cultured on Schaedler medium, Columbia agar with 5 % defibrinated sheep blood and Mannitol-salt, MacConkey, Sabouraud agars (37 °C for at least 48 h). The cultures were prolonged up to 14 days for slow-growing and fastidious microorganisms. The identification of isolated strains was based on morphology of the colonies, microscopic examination, and biochemical tests (microbiological analyzer Vitek 2 compact, bioMérieux, Marcy L’Etoile, France). Removed prefabricated spacers were subjected to sonication and molecular detection as previously described [13].

### Histopathological tests

Soft tissue surrounding the spacer and periprosthetic interface membrane were taken for histopathological testing. The outcomes were recorded according to Krenn and Morawietz classification [14].

## Results

### C-reactive protein results

CRP (C-reactive protein) was significantly elevated in 1 of 13 cases. In this case (patient nr 11) the failure after 2-years observation was noted. In remaining two cases CRP level was minimally elevated without failures in follow up. In the group with no elevated CRP level (10 patients) we noted 4 culture-positive cases (patients 4, 8, 9, 13) and only in case nr 13 it was related to failure.

### Microbiological results

#### Intraoperative specimens after first stage

The result of tissue cultures in the first stage of surgery were positive in 7 cases with the growth of *Micrococcus sp., Streptococcus viridans, Escherichia coli, Enterococcus faecalis, Enterococcus faecium, Enterobacter cloacae, Acinetobacter baumannii*, and *Staphylococcus aureus*. Six out of 13 studied cases did not reveal the growth of microorganisms and no failures in 2 years follow-up were noted.

#### Joint fluid culture

The negative joint fluid culture results before second-stage revision were obtained in all 13 cases.

#### Intraoperative specimens during 2^nd^ stage

In 2 cases (15 %) a positive culture revealed the growth of CNS (Coagulase-Negative Staphylococci)—*Staphylococcus epidermidis*. In case nr 8 this culture was not concordant with sonicate fluid negative culture and no failure was observed. In contrast to this case, in the case of patient nr 13 the positive culture of tissue sample was concordant with the positive culture of sonication fluid and a failure was noted. The culture results of intraoperative specimens were negative in remaining 11 cases.

#### Sonicate culture

In three cases cultures of sonicate liquid revealed the presence: in one case *S. epidermidis* (patient nr 13) and in two cases *Ralstonia pickettii* (patients nr 4 and 9). Failure was noted only in case nr 13 with the *S. epidermidis* growth. In remaining two cases of *R. pickettii* growth no failure was observed. We found 77 % compatibility (10 cases) between culture results of synovial fluid obtained through the joint aspiration and sonicate fluid obtained from components of prosthesis, and 69 % compatibility (9 cases) when cultures of intraoperative specimens and sonicate fluid results were analyzed. The sonicate cultures were negative in 10 cases (Table 1).

**Table 1:** Clinical details of patients

Patient	Affected joint	CRP before 2^nd^ stage	Time between 1^st^ and 2^nd^ stage (days)	Treatment	Culture result - 1^st^ stage	Culture results - 2^nd^ stage			Molecular identification Bacteria identified by 16S rRNA gene sequencing	Followup (mean, 32 months; range, 25–36 months)
					Intraop. specimen	Preop. samples (joint fluid)	Intraop. specimen	Sonicate		
1	H	<5	263	Restoration Stryker	negative	negative	negative	negative	*Geobacillus stearothermofilus, G. vulcani*	healed
2	H	<5	150	Centrament Aesculap	negative	negative	negative	negative	*Lactobacillus jensenii, L. acidophilus, L. fornicalis*	death
3	K	<5	146	Scorpio TS Stryker	negative	negative	negative	negative	*Pseudomonas aeruginosa, P. resinovorans*	healed
4	K	<5	90	Scorpio TS Stryker	negative	negative	negative	*Ralstonia pickettii*	*Novosphingobium nitrogenifigens, N. hassiacum, Bradyrhizobium japonicum, B. liaoningense,*	healed
5	H	6,4	145	Restoration Stryker	negative	negative	negative	negative	*Klebsiella pneumoniae*	healed
6	K	<5	170	Scorpio TS Stryker	negative	negative	negative	negative	*Klebsiella pneumoniae*	healed
7	K	6	184	Scorpio TS Stryker	*Micrococcus sp.*	negative	negative	negative	*S. lugdunensis, S. hominis*	healed
8	K	<5	88	Scorpio TS Stryker	*Streptococcus viridans*	negative	*S. epidermidis*	negative	*Corynebacterium ureicelerivorans, C. mucifaciens*	healed
9	K	<5	150	Scorpio TS Stryker	*E.coli*	negative	negative	*Ralstonia pickettii*	*Rubrobacter xylanophilus, Clostridium saccharoperbutylacetonicum*	healed
10	K	<5	150	Scorpio TS Stryker	*Enterococcus faecalis*	negative	negative	negative	*Tuberibacillus calidus, Bacillus algicola*	healed
11	K	27,1	140	Scorpio TS Stryker	*Acinetobacter baumani Enterobacter cloacae*	negative	negative	negative	*negative*	failure: prolonged antibiotic therapy (recurrent joint effusion)
12	H	<5	135	Restoration Stryker	*Enterococcus faecium*	negative	negative	negative	*Brevibacterium ravenspurgense, B. paucivorans*	healed
13	K	<5	180	Arthrodesis ChM plate	*Staphylococcus aureus*	negative	*S. epidermidis*	*S. epidermidis*	*Acinetobacter johnsonii, A. parvus*	failure: prolonged antibiotic therapy (prolonged wound healing)

*H* hip, *K* knee

### Molecular detection

The presence of bacterial DNA (deoxyribonucleic acid) was confirmed with molecular testing in 92 % of patients with negative synovial fluid cultures. In most samples, positive PCR results revealed presence of 2 or more different opportunistic bacteria (eg, *S. epidermidis*, *Klebsiella pneumoniae, Acinetobacter* spp*., Pseudomonas* spp., *Lactobacillus* spp.) and most of them belongs to human or environmental microflora with low virulence. All results are presented in Table 1.

### Histopathological test

The results of histopathological tests revealed the presence of infection features (type II) in all cases. Type II is defined as the presence of activated fibroblasts, proliferation of small blood vessels, edema, and inflammatory infiltration of neutrophilic granulocytes in the periprosthetic membrane.

### Follow-up

In 10 patients no failures were noted: lack of any clinical features of infection, radiological findings of implants loosening, increasing laboratory markers, and prolonged antibiotic therapy. The clinical examination revealed good outcomes. Failure at final follow-up was recorded in 2 (16.6 %) patients.

**Fig. 1:**
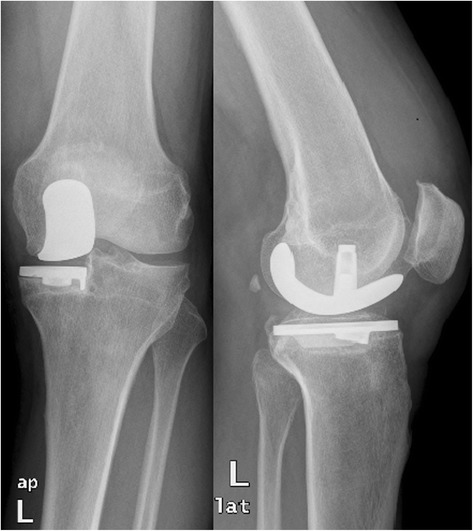
X-rays (AP and lateral view) of loosened implants of unicompartmental knee arthroplasty

**Fig. 2:**
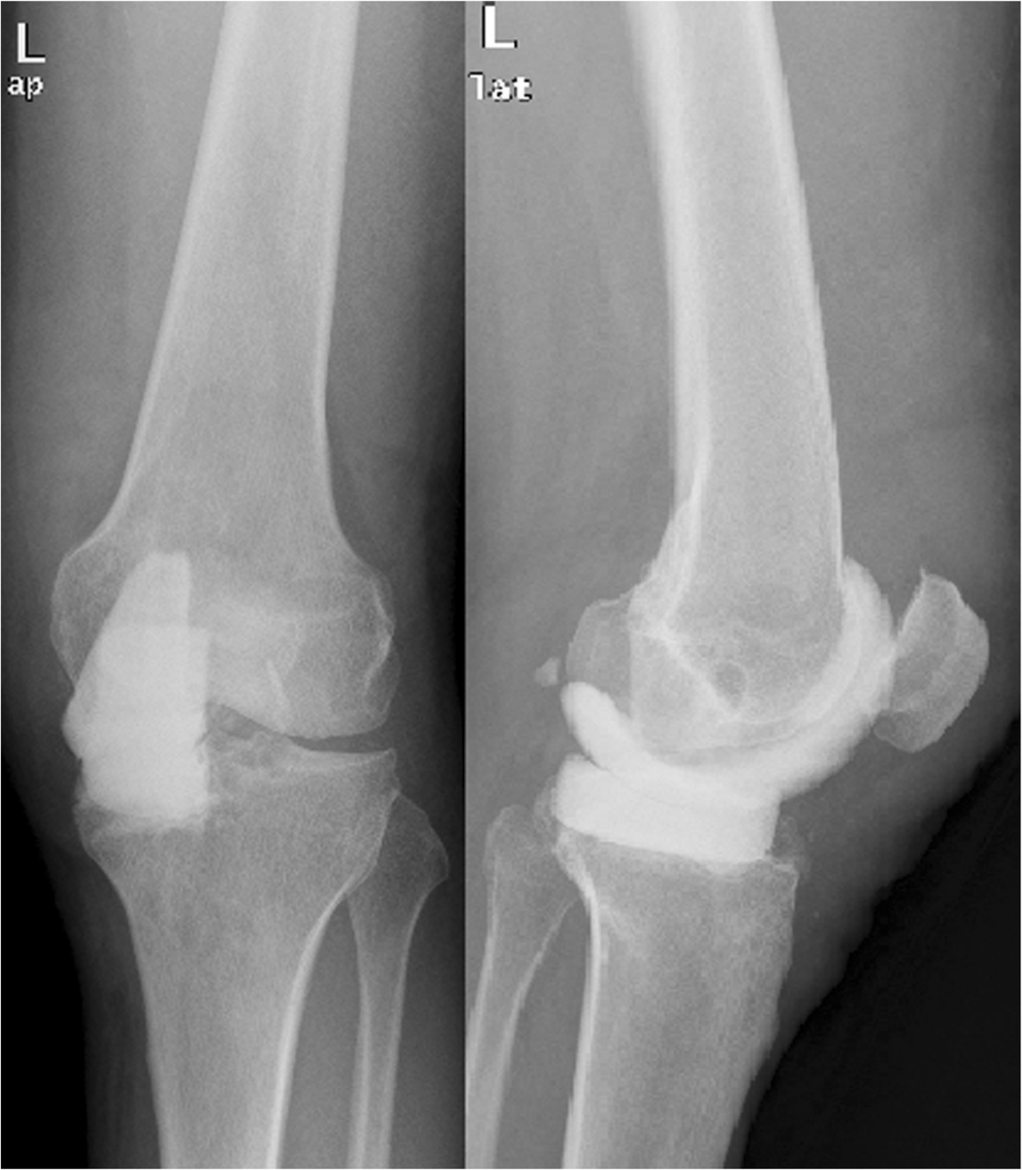
X-rays (AP and lateral view) after 1^st^ stage of two-stage revision arthroplasty (Spacer implantation)

**Fig. 3:**
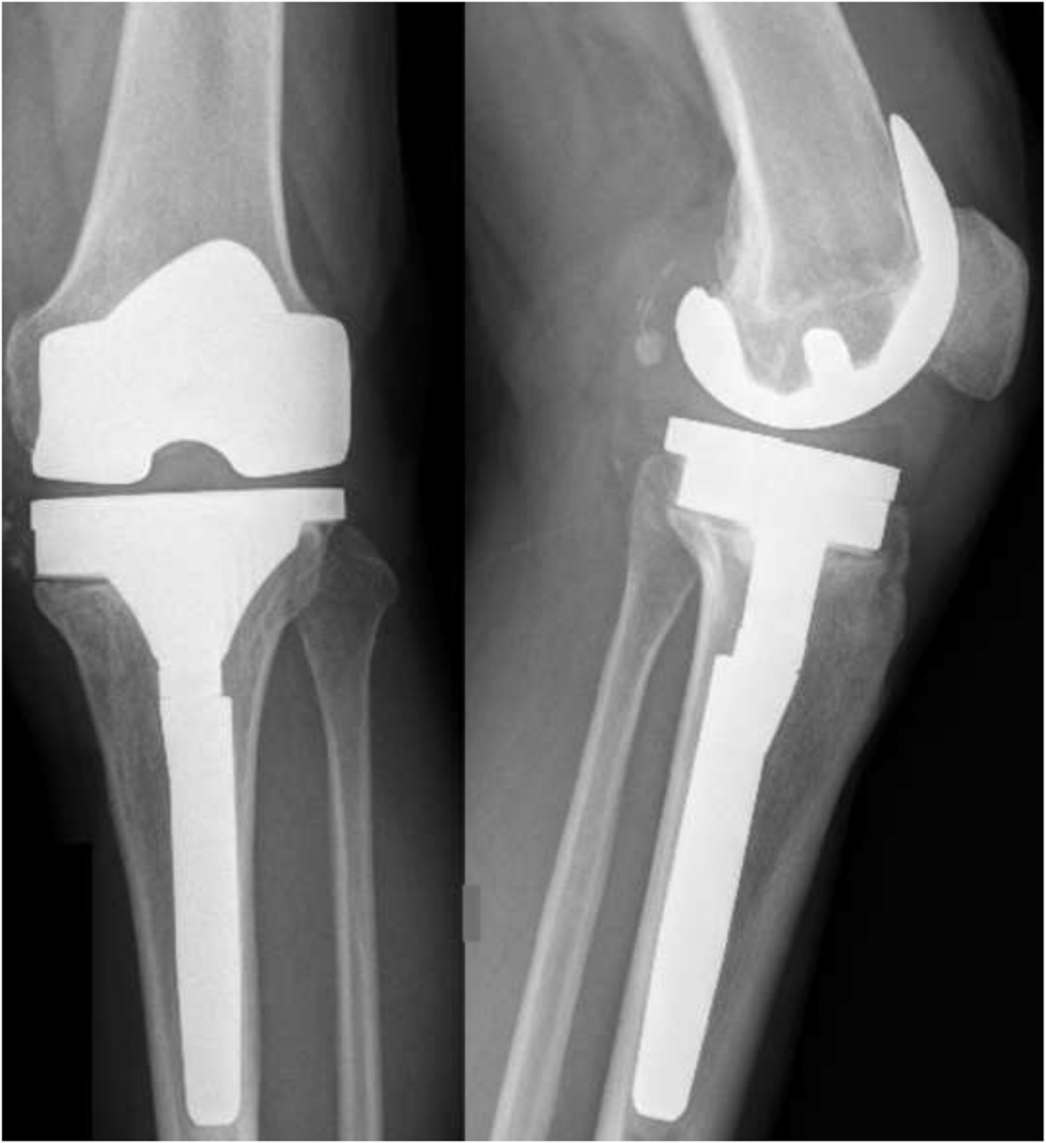
X-rays (AP and lateral view) of knee joint after second stage of revision arthroplasty (revision implants)

**Fig. 4:**
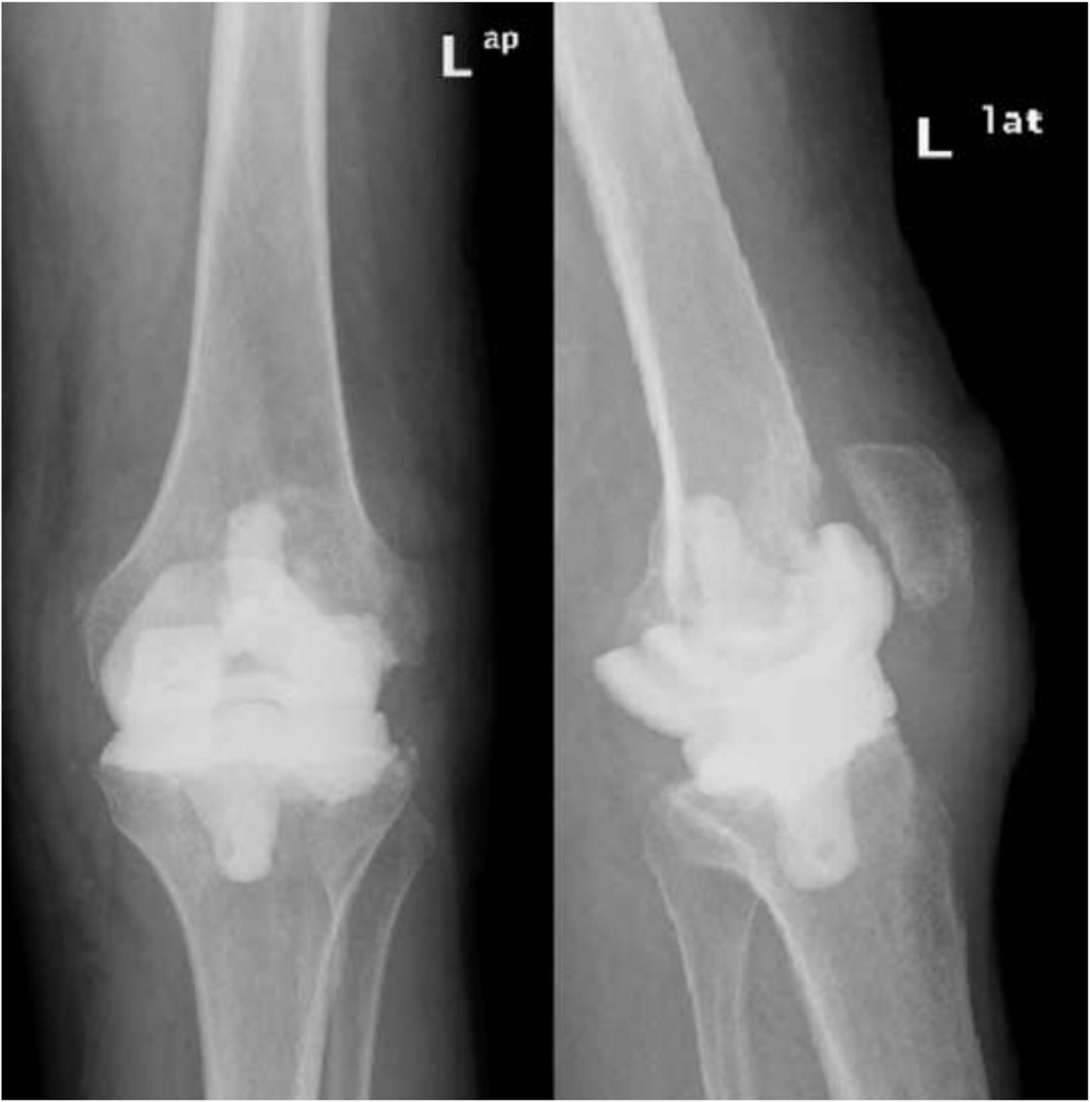
X-rays (AP and lateral view) after 1^st^ stage of surgery treatment with implantation of knee Spacer

**Fig. 5:**
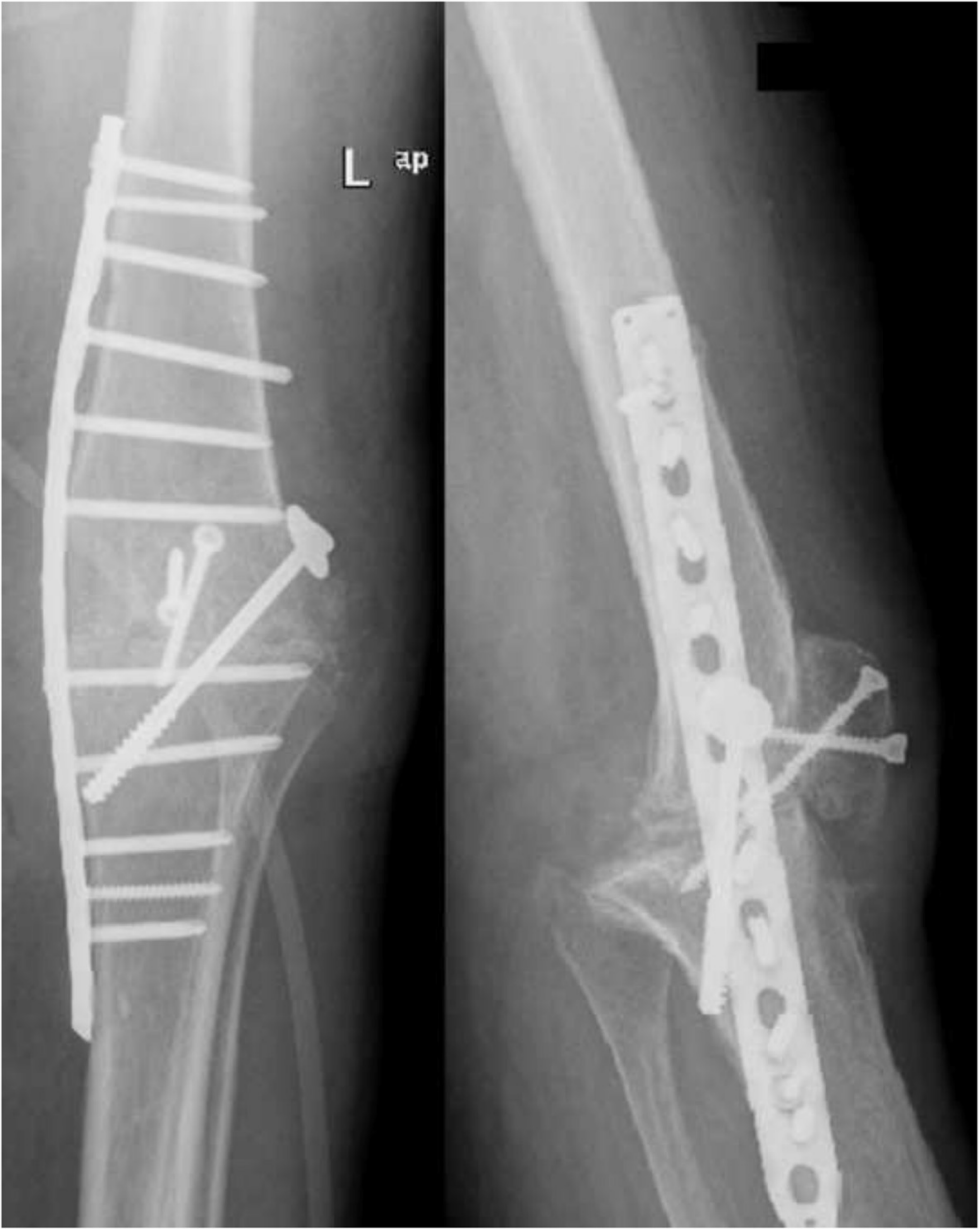
X-rays (AP and lateral view) after removal of Spacer and arthrodesis of left knee joint

In patient nr 11 (Figs. 1, 2 and 3), the clinical data revealed the periodic effusion without persistent pain, the presence of MSSE (Methicilin Sensitive *Staphylococcus epidermidis*) in 1/3 arthrocentesis performed in the early postoperative period. A minimal radiolucency was noticed under the tibial component, however no other radiological feature of implants loosening was observed. This case was not assessed as implant loosening (Fig. 3). However, in 2 years observation infection of *S. epidermidis* occurred. Targeted antimicrobial therapy was administered. For these reasons this case was assessed as a failure. The second recognized failure (patient nr 13; Figs. 4 and 5) was also due to infection with *S. epidermidis.* The decision about second-stage revision surgery was based on the medical history of the patient: knee joint infection many years ago, clinical signs of infection after primary knee joint arthroplasty and the growth of *S. aureus* from intraoperative tissue samples taken during the first-stage revision surgery. This patient finally underwent arthrodesis of the knee joint due to general medical condition and the high risk of reinfection. Prolonged wound healing, the positive culture results from intraoperative specimens and sonicate fluid (*S. epidermidis*) were the reason for long-term antibiotic therapy in this case. We qualified this case as a failure with the recognition of infection with *S. epidermidis*.

One of the 13 patients was lost to follow-up due to death, which did not occur perioperatively or within 90 days postoperatively, and arguably was not related to the arthroplasty or infection (Table 1).

## Discussion

We compared the intraoperative specimens, joint fluid, and sonicate cultures with sequencing results. We found that the diagnosis of “treated” PJI does not exclude the presence of bacteria or bacterial DNA on the surface of prefabricated spacers. Our literature search revealed few studies identifying microorganisms on the surface of removed antibiotic-loaded cement spacers using both sonication and 16S rRNA (ribosomal ribonucleic acid) gene sequencing.

Two-stage exchange arthroplasty is a surgical strategy for PJI treatment. It is considered as a standard procedure for surgical treatment of late PJI, with treatment success estimated at 73–89 % [7, 15]. The limited sensitivity and specificity of standard bacterial culture techniques may limit their ability to detect the adherent bacteria responsible for PJI [16, 17]. In the present study we cultured the sonication fluid of removed spacers to confirm the eradication of infection. This process considerably enhances culture sensitivity and represents a cheap, easy, accurate, and sensitive diagnostic modality compared to periprosthetic tissue cultures. The technique is simple and can be performed in most microbiology laboratories [4, 17].

Recent studies reported 14.5 to 29 % positive sonicate cultures of the removed spacers and noted that an infection of the cement spacer is associated with poor clinical outcome [16, 18, 19]. The incidence of reinfection after two-stage exchange arthroplasty has been estimated at 10–31 % [7, 16, 20]. Our study revealed the presence of microorganisms on the surface of prefabricated spacers in 2 cases in intraoperative specimens (15 %) and in 3 cases after sonication (23 %). The samples after sonication in 1 case revealed *S. epidermidis—*a proven etiological agent of infection related with biomaterials, and in the 2 other cases—*R. pickettii*. This Gram-negative, non-fermenting bacteria, able to live in aquatic environment, in low-nutrient conditions, and in hospital water supplies, is more often indicated as the cause of osteomyelitis, meningitis, and sepsis, especially in patients with risk factors. Ryan and Adley described the bacteria from the *Ralstonia* genera as emerging global opportunistic pathogens and considered them to be as important as severe infections causative agents, such as *Acinetobacter baumannii*, *Pseudomonas aeruginosa*, *Burkholderia cepacia,* and *Stenotrophomonas maltophilia* [20]. These bacilli were found in intravascular catheters and other biological materials [21]. The positive culture of *R. pickettii* should be interpreted in the aspect of the clinical findings and level of inflammatory mediators. On the other hand, sonication findings should be considered in the aspect of false positive results due to prostheses contamination at the operating room. Contamination is possible in the region of the surgical site, during the collection and handling of the samples, particularly if some fluids are added to the sample and, rarely, during specimen processing in the laboratory. Further studies concerning *Ralstonia* pathogenicity are required.

The sonication procedure and PCR followed by sequencing were positive in 92 % of studied cases. PCR of sonication fluid from removed spacers revealed the variety of species in our study. Additional pathogens were detected in other studies [22, 23]. For the microbiologic diagnosis of prosthetic joint infection, PCR of sonicated samples is more sensitive than tissue culture [24]. However, identification of bacterial DNA in PCR assay does not confirm the presence of live bacteria. The identification of etiological agents but also contaminating factors is possible due to the high specificity of PCR techniques. The main limitations of PCR relate to inherent problems with contamination and sensitivity. Contamination arises from bacterial DNA present in PCR reagents or inadvertently introduced during the collection and handling of the sample. This is because sterile fluid may nonetheless contain microbial DNA. Contaminants detected with the PCR (false-positive results) belong to the same type of pathogens as the microorganisms causing low-grade PJI, making the distinction of true-positive and false-positive PCR results difficult [25]. In this study we identified DNA of various bacterial species. Some of them (eg, *Klebsiella pneumoniae* and *Pseudomonas aeruginosa*) cause serious, life-threatening diseases*,* and others occur in the human environment or food (eg, *Lactobacillus* spp. *Brevibacterium* spp., and *Corynebacterium* spp.). Many of the species are described as etiological agents of infection, especially in patients with immunodeficiency [26, 27].

Our study estimated the average time between surgeries to approximately 5 months. When analyzing other studies, we noticed that the mean time between stages was comparable [6]. Based on several studies, we can conclude that spacers should be left at the PJI site no longer than 6 months, but even 3 months could be enough for eradication of bacteria [28, 29]. The new IDSA (Infectious Diseases Society of America) Guideline of PJI recommends the period 6–14 weeks from resection arthroplasty to reimplantation (4–6 weeks of antimicrobial therapy and 2–8 weeks of antibiotic-free period). This delayed reimplantation has been noted to be highly successful [30]. This period of local antibiotic elution to the periprosthetic infection site is considered as sufficient, and the colonization of spacer surface with new microorganisms can be prevented.

In our study histopathology of periprosthetic tissues revealed the infectious type (type II) in all cases, pointing to the PJI. The periprosthetic membrane is an ideal material for characterizing the type of inflammation by histopathology and provides valuable evidence for the underlying cause of failure [14].

We described treatment failure in 2 out of 13 patients at 2.5-year follow-up. In 1 of them (patient 13), intraoperative and sonicate cultures were positive. Some studies reported that the recurrence of infection may be more frequent if caused by resistant bacteria such as methicillin-resistant *S. aureus* (MRSA) and methicillin-resistant *S. epidermidis* (MRSE) or in cases requiring multiple prior open procedures [7, 31]*.* Nelson found 9 (50 %) patients with reinfection out of 18 with positive sonicate cultures [32]. Followed by recommendation of some authors removal of prosthesis or arthrodesis can be performed in cases of serious comorbidity or unacceptable to the patient repeated surgery or which seem deemed unsafe [33]. In most cases (patients 1–12) patients underwent two-stage exchanging arthroplasties with implantation of the revision implants.

We had 1 death out of 13 patients. Another study reported no patient deaths within 3 months after the operation and 16 % overall mortality rate [34].

Our previous as well as the present survey confirmed the presence of bacterial DNA on both, the removed loosed implants due to aseptic loosening and prefabricated cement spacers used in two-stage revision arthroplasties [13].

In conclusion, this study confirmed that sonication followed by PCR and sequencing improves bacterial detection. The high rate of identified bacterial DNA in presumed healed PJI is not considered in our study as a cause of failures. The prolonged elevated CRP level in one case, and previous infection of operated joint in the second case were the reasons for failures. Thus, the treatment strategy should be considered in the light of history of the disease, comorbidities, clinical and laboratory results. Prolonged period between two stages of revision arthroplasty could be the reason for colonization of spacer surfaces with new microorganisms, especially dangerous for patients with immunodeficiency. Taking into consideration our results and observations of other authors, the shortening of time interval between stages to 6–14 weeks is beneficial [28–30].

### Study limitations

The main limitations of our study are the small sample size and the short period of observation. Larger studies are needed to confirm these results before we could recommend them for wider use. Nevertheless, the results are very promising.

## Conclusion

Firstly, the lack of clinical signs of infection, negative culture results of pre- and intraoperative samples do not exclude existence of bacteria on the surfaces of pre-formed antibiotic-loaded spacers used in two-stage exchange arthroplasties. Secondly, the positive results of sonication and molecular tests should be interpreted as real pathogenicity factors in the light of the clinical, microbiological and histopathological data, especially for patients with immunodeficiency. Finally, more attention should be paid to reimplantation of spacers in patients without clinical symptoms of infection with prolonged elevated level of CRP and in cases of prior infectious process of operated joint. Above conclusions state the clinical significance of our results and should be useful for clinicians.

## Abbreviations

CNS: Coagulase-Negative Staphylococci
CRP: C-reactive protein
DNA: Deoxyribonucleic acid
IDSA: Infectious Diseases Society of America
MRSA: Methicilin resistant *Staphylococcus aureus*
MSSE: Methicilin Sensitive *Staphylococcus epidermidis*
PCR: Polymerase chain reaction
PJI: Prosthetic joint infection
rRNA: Ribosomal ribonucleic acid
